# Detection of SARS-CoV-2 in the sewerage system in Tunisia: a promising tool to confront COVID-19 pandemic

**DOI:** 10.2217/fvl-2021-0050

**Published:** 2021-10-14

**Authors:** Habib Jmii, Hakima Gharbi-Khelifi, Raouia Assaoudi, Mahjoub Aouni

**Affiliations:** 1 ^1^Laboratory of Transmissible Diseases & Biologically Active Substances (LR99ES27), Faculty of Pharmacy of Monastir, University of Monastir, Monastir, Tunisia; 2 ^2^Faculty of Sciences and Techniques of Sidi Bouzid, University of Kairouan, Tunisia

**Keywords:** SARS-CoV-2, Tunisia, virus monitoring, wastewaters

## Abstract

**Aim:** The current study undertaken in Tunisia examines the use of wastewaters to monitor SARS-CoV-2 circulation. **Materials & methods:** Viral genetic materials collected in wastewaters during two different periods (September–October 2020 and February–April 2021) were concentrated using the adsorption-elution method. SARS-CoV-2 genes were researched by real-time PCR. **Results:** During the first period of the study, viral RNA was detected in 61.11% of the analyzed samples collected from Monastir city with a rate of 88.88% for raw wastewaters and 33.33% for treated wastewaters. Then, during the second period of the study, the quantitative analysis of wastewaters collected from seven governorates showed the presence of viral RNA among around 25% of them with variable RNA loads. The increased amounts of viral RNA detected in wastewaters were accompanied by an increase in the number of COVID-19 patients in Tunisia. **Conclusion:** Our results emphasize the importance of sewage survey in SARS-CoV-2 tracking.

## Graphical abstract

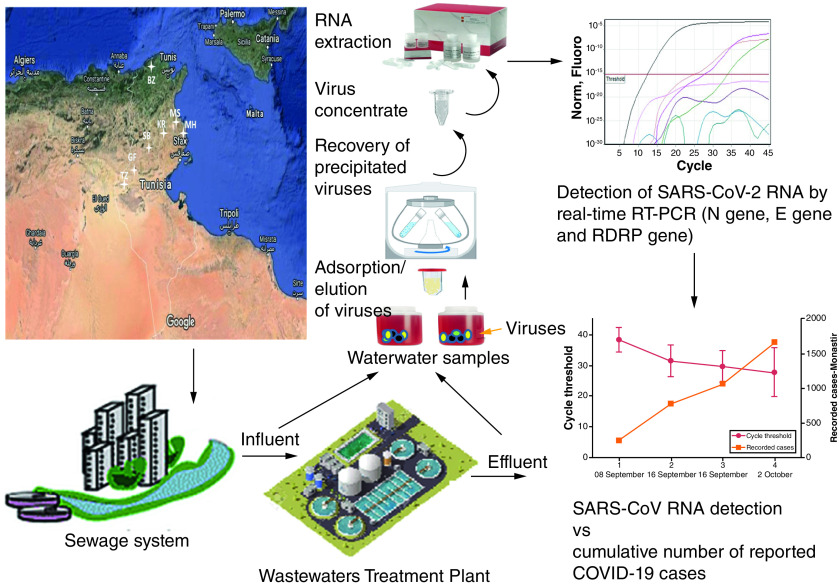

Since its emergence in December 2019 in the Chinese city of Wuhan, SARS-CoV-2 continues to spread and practically all the countries of the globe are affected by the pandemic of COVID-19. The number of confirmed cases and deaths of COVID-19 is inceasingly growing. By 16 September 2021, a total of 218 countries and territories around the world have reported 227,471,677 confirmed cases and 4,677,080 deaths according to the WHO.

Surveillance of COVID-19 pandemic is essentially based on clinical epidemiology since healthy individuals can be directly infected by inhaling and mucosa-contacting respiratory droplets/aerosols emitted from individuals carrying the virus. However, the contaminated matrices, including surfaces, fixed structures, medical apparatus, foods, beverages etc., may also play a pivotal role in the indirect transmission routes of SARS-CoV-2. Furthermore, the detection of SARS-CoV-2 in sewage has raised concerns about the fecal–oral transmission and infection through the urban water cycle system especially where treated wastewaters are reused in agriculture [1,2]. The other factors that can also affect the transmission dynamics of the COVID-19 pandemic include differences in social habits and interactions, and environmental conditions and economic aspects [1]. In a study conducted in 2020 by Coccia and his colleagues in Italy, it has been demonstrated that air pollution fosters viral transmission [3]. In addition, commercial exchanges can be a source of viral transmission as suggested by another study in Italy demonstrating that the high incidence of infection cases detected in the northern part of the country in early March 2020 is most likely related to the strong commercial/economical relationship between China and northern Italy which would be the origin of COVID-19 initial diffusion phase in Italy [1]. Thus, an inclusive strategy including medical and non-medical actors should be adopted for the prevention and control of epidemics.

The clinical epidemiology survey of the COVID-19 pandemic is arduous, especially in developing countries where resources are not enough to test all symptomatic cases and the persons who had contact with them. In addition, asymptomatic, presymptomatic and pauci-symptomatic cases of coronavirus infections could be an important source of contagion making determining the true scale of viral circulation in a community difficult [4]. Hence, finding tools for a massive screening and a fast detection of coronavirus to halt its dissemination is challenging. Wastewater-based epidemiology (WBE) could be a reliable strategy for preventive tracking and diagnosing of COVID-19 across communities. The presence of SARS-CoV-2 in wastewaters has been found in several recent studies conducted in many countries namely but not exhaustively the USA, UK, Netherlands, France, Spain, Italy, Germany, Japan, India, Australia, Brazil, United Arab Emirates, Iran and South Africa [5–20]. Some of these studies have reported the occurrence of SARS-CoV-2 RNA in wastewaters before the registration of the first confirmed cases by health authorities [7,10,11,16]. Importantly, WBE seems to be an efficient tool in the early detection of the circulation of new variants of SARS-CoV-2 in a population (reviewed in [21]), to evaluate the success of lockdown measures in controlling COVID-19 [6,8,22], and WBE can be also adapted for application in the orientation of the mass vaccination program according to the prevalence of the pandemic (reviewed in [23]). In Tunisia, after winning the battle against the novel Coronavirus during the first wave (March–May 2020), the pandemic has rebounded strongly by the beginning of August. The number of positive cases has considerably increased, and the situation became worrying. In this study, we report the results of the screening for SARS-CoV-2 RNA presence in sewage samples collected from 14 wastewater treatment plants (WWTPs) located in seven different regions, from the north to the south of the country (Figure 1 & Table 2). This work is aimed to back-up the efforts of health authorities to monitor coronavirus spreading, identifying the hotspots, and helping in pandemic management in the involved regions. Furthermore, this work is intended to get insights about the efficacy of treatments employed in the studied WWTPs against SARSCoV-2 particularly when wastewater effluents are reused for agriculture.

**Figure 1.**
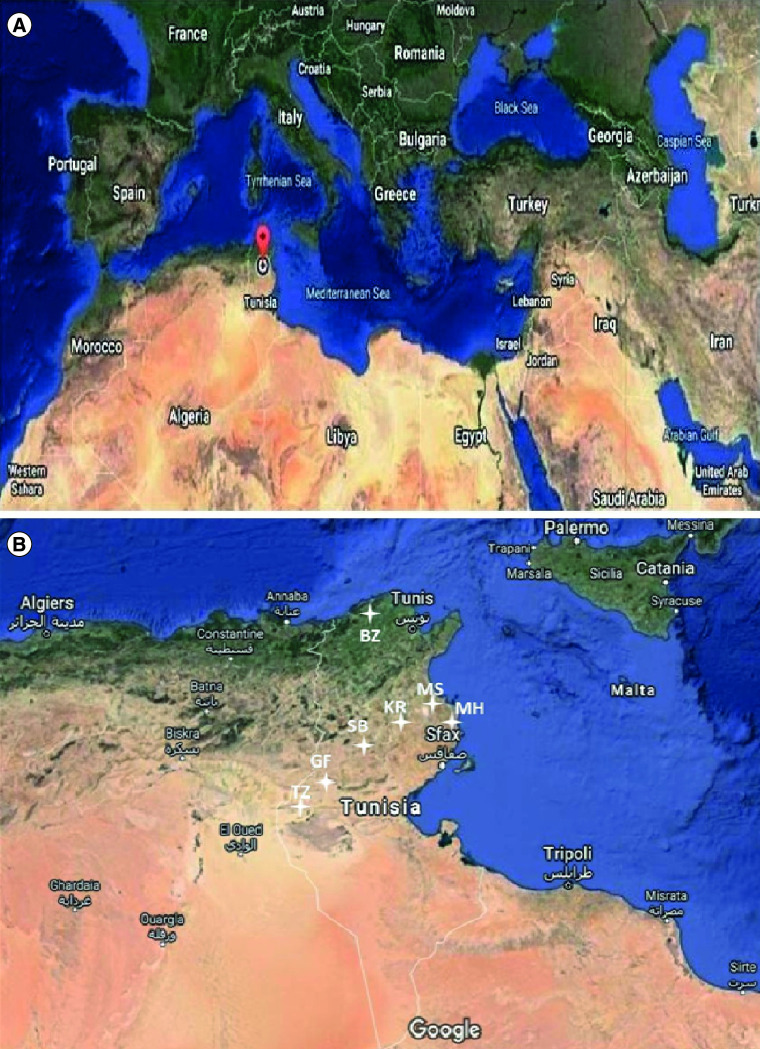
Localization of Tunisia with the different regions selected for wastewaters sampling.

## Materials & methods

### Sampling

During the first period of our study, three WWTPs located in different municipalities of the region of Monastir (center-east of Tunisia, Figure 1) were studied. Sampling was carried out weekly from 8 September 2020 to 2 October 2020. Samples were collected in two-liter plastic containers, kept at 4°C upon arrival, and concentrated within 24 h. The choice of WWTPs was based on the nature of the treatment used in sewage treatment, the districts connected to the WWTP and the destination of the effluent treated wastewaters. Sampling sites, wastewaters processes, discharge points and the reuse of treated wastewaters data are indicated in the Table 1.

**Table 1.** Data about wastewater treatment plants involved in the study with results of real-time amplification of SARS-CoV-2 RNA in wastewaters collected from the region of Monastir during the period between 8 September 2020 and 2 October 2020.

Sampling site	Wastewater treatment processes	Discharge points	Reuse	Date of sampling	Water sample	Mean of Ct values		
						Gene		
						*N*	*E*	*RDRP*
WWTP Sahline	Oxidation ditch extended aeration activated sludge	River Oued Hamdoun to sea	Irrigation of the golf course PalmLynk	08-09-2020	Influent	-	-	-
				08-09-2020	Effluent	-	-	-
				16-09-2020	Influent	25.98	-	22.48
				16-09-2020	Effluent	-	-	-
				22-09-2020	Influent	26.87	31.4	26.12
				22-09-2020	Effluent	37.64	-	-
WWTP Wardenine	Activated sludge	Oued lGuelta	Irrigation of different agriculture cultures	08-09-2020	Influent	-	-	30.30
				08-09-2020	Effluent	-	-	-
				16-09-2020	Influent	33.14	31.32	34.67
				16-09-2020	Effluent	-	-	-
				22-09-2020	Influent	32.75	36.12	30.5
				22-09-2020	Effluent	-	-	-
WWTP Frina	Activated sludge	Sea	Irrigation of the golf course PalmLynk	16-09-2020	Influent	29.11	33.12	33.69
				16-09-2020	Effluent	-	-	-
				22-09-2020	Influent	28.56	35.30	19.5
				22-09-2020	Effluent	34.65	40.00	34.02
				02-10-2020	Influent	28.56	35.30	19.5
				02-10-2020	Effluent	34.65	40.00	34.02

WWTP: Wastewater treatment plant.

Afterward, during the second period of our study, we have broadened the extent of our research to cover seven regions throughout the country (Figure 1). We received 31 raw wastewater samples during the period from 24 February 2021 to 4 May 2021 (Table 2).

**Table 2.** Quantitive analysis for the presence of the SARS-CoV-2 RNA in wastewaters.

Sample	Governorate	Locality	Date of reception	Type of received wastewaters	Quantity of analyzed wastewaters (l)	Absence/presence of viral RNA	Number of gene *N* fragment copies (10^3^ copies/100 ml)	
							N1	N2
1	Kairouan	Kairouan	24/02/2021	Influent	2	-	-	-
2	Kairouan	Haffouz	24/02/2021	Influent	2	-	-	-
3	Tozeur	Tozeur	24/02/2021	Influent	2	-	-	-
4	Monastir	Sahline	24/02/2021	Influent	2	+	14.22	1.98
5	Monastir	Frina	24/02/2021	Influent	2	-	-	-
6	Monastir	Wardenine	24/02/2021	Influent	2	-	-	-
7	Gafsa	El Aguila Gafsa	24/02/2021	Influent	2	-	-	-
8	Gafsa	Metlaoui	24/02/2021	Influent	2	+	-	-
9	Mahdia	Mahdia	24/02/2021	Influent	2	-	-	-
10	Mahdia	Echebba	24/02/2021	Influent	2	+	5.58	12.6
11	Mahdia	Gssour Essef	24/02/2021	Influent	2	-	-	-
12	Bizerte	Sidi Ahmed	24/02/2021	Influent	2	+	-	-
13	Bizerte	Menzel Bourguiba	24/02/2021	Influent	2	-	-	-
14	Sidi Bouzid	Sidi Bouzid	24/02/2021	Influent	2	+	0.42	0.28
15	Kairoun	Kairoun	14/04/2021	Influent	2	-	-	-
16	Kairoun	Haffouz	14/04/2021	Influent	2	-	-	-
17	Sidi Bouzid	Sidi Bouzid	14/04/2021	Influent	2	-	-	-
18	Tozeur	Tozeur	14/04/2021	Influent	2	-	-	-
19	Bizerte	Sidi Ahmed	14/04/2021	Influent	2	-	-	-
20	Bizerte	Menzel Bourguiba	14/04/2021	Influent	2	-	-	-
21	Mahdia	Mahdia	14/04/2021	Influent	2	-	-	-
22	Mahdia	Gssour Essef	14/04/2021	Influent	2	+	0.18	0.24
23	Mahdia	Echebba	14/04/2021	Influent	2	-	-	-
24	Monastir	Sahline	14/04/2021	Influent	2	-	-	-
25	Monastir	Frina	14/04/2021	Influent	2	+	0.45	0.30
26	Monastir	Wardenine	14/04/2021	Influent	2	+	4.25	0.8
27	Mahdia	Mahdia	27/04/2021	Influent	2	-	-	-
28	Mahdia	Ksour essef	27/04/2021	Influent	2	-	-	-
29	Mahdia	Chebba	27/04/2021	Influent	2	-	-	-
30	Gafsa	Gafsa	04/05/2021	Influent	2	+	59.94	5.31
31	Gafsa	Metlaoui	04/05/2021	Influent	2	+	0.84	0.33

### Viral genetic materials concentration

Viral genetic materials were concentrated by the adsorption-elution method using aluminum hydroxide and beef extract as described by [9] with minor modifications. The raw wastewater samples (2 L) were subjected to a coarse filtration, this step was omitted for treated wastewaters (effluents). Then, the filtered wastewaters were re-filtered through 0.45 μm membranes. Then, these membranes were cut and placed in 250 ml polyropylene copolymer (PPCO) centrifuge bottles. An amount of 100 ml of the obtained filtrate were added to the membrane pieces and vigorously vortexed to detach the viral particles stuck to the membranes. PPCO bottles were centrifuged at 2000 rpm for 5 min and supernatants were collected. Afterward, 100 ml of collected supernatants were placed in a PPCO centrifuge bottle, pH was adjusted to 6.0 and aluminum hydroxide solution (0.9 N) was added to the wastewaters sample (1:100). The pH was readjusted to 6.0 and the sample was shaken using an orbital shaker at 150 rpm for 15 min at room temperature to allow virus absorption, and precipitates were recovered by centrifugation at 1700 g for 20 min. Then, viral genetic materials elution was carried out by resuspending the obtained pellet in 10 ml of 3% beef extract (pH 7.4). Viral suspension was then transferred in 50 ml PPCO centrifuge tubes and shaken for 10 min at 150 rpm Afterward, viral genetic materials were recovered by centrifugation at 1900 g for 30 min and the pellet was re-suspended in 1 ml of phosphate-buffered saline. The obtained concentrates were aliquoted and conserved at -80°C until being used.

### Viral RNA extraction

Before proceeding to RNA extraction, viral concentrates were spiked with 10 μl of exogenous viral RNA which is composed of MS2 bacteriophage genome (provided in the kit used in real-time PCR experiments). This internal control material enables to verify the efficiency of RNA extraction, reverse transcription, PCR steps to demonstrate proper specimen processing, and the absence of amplification inhibitors. RNA was extracted from 300 μl of concentrates using the RNeasy PowerWater Kit (Qiagen) according to the manufacturer’s instructions. The RNA was eluted in 50 μl of RNase-free water, aliquoted and conserved at -80°C until being used for viral RNA detection.

### Qualitative & quantitative detection SARS-CoV-2 RNA

During the first period of the current study, qualitative analysis for the presence of SARS-CoV-2 RNA in wastewaters was performed using the Allplex 2019-nCoV kit (Seegene, Seoul, South Korea) allowing the detection of three viral targets: the *E*, *N* and *RdRp* genes by real-time reverse transcriptase polymerase chain reaction (RT-PCR). Manufacturer’s instructions have been followed in preparation for real-time PCR and thermal cycling conditions. Reaction mix (25 μ) consisted of 5 μl of 2019-nCoV MOM containing primers and probes, 5 μl of 5X real-time One-step buffer, 5 μl of RNase-free water, and 2 μl of real-time one-enzymes. The thermal cycling conditions were as RT at 50°C for 20 min, preheating at 95°C for 15 min, and 45 cycles of amplification at 94°C for 15 s and 58°C for 30 s. Each sample was analyzed in triplicate and every real time RT-PCR assay included negative (RNase-free water) and positive controls (SARS-CoV-2 RNA provided in the kit). In addition, the positive signal for the internal control indicates that all steps performed from RNA extraction to the amplification of viral RNA were successful. The threshold cycle was set to 40 for all target genes and samples which were found positive for at least the *N* or the *RdRp* genes were considered positive as recommended by the manufacturer. In case of positivity for only the *E* gene, the sample is considered uncertain.

During the second period of our study, we performed a quantitative analysis for SARS-CoV-2 RNA using the QuantiTect virus Kit (Qiagen, Hilden, Germany) enabling a one-step quantitative detection of the viral RNA targets. We targeted the gene *N* of SARS-CoV-2, two fragments of this latter were amplified namely *N1* and *N2*. CDC Primers/probes were used in the RT-PCR reaction at the concentration recommended by the CDC. Each sample was analyzed in triplicate and every real time RT-PCR assay included negative (RNase-free water) and positive controls (SARS-CoV-2 RNA, Qiagen). The threshold value was set to 0.03 and the cycle threshold was set to 40. The standard curve was constructed using single stranded RNA fragments of SARS-CoV-2 containing the target region: gene *N* (Joint Research Centre, EURM-019).

## Results

### SARS-CoV-2 RNA detection in raw & treated wastewaters

During the first period of our study between 8 September 2020 and 2 October 2020, nine influent and nine effluent wastewater samples were investigated for the presence of SARS-CoV-2 RNA. Samples were considered positive for Ct below 40 for at least one of the two targeted genes *N* and *RdRp* as done in the previous studies in which the threshold cycle for positivity was set to 40. Out of the 18 samples analyzed, 11 were positive for viral RNA (61.11%) (Table 1). Among the 11 positive samples, ten (90.90%) were positive for the gene *N*, six (54.54%) were positive for the gene *E* and ten (90.90 %) were positive for the gene *RdRp*. The highest number of positive samples was recorded in samples collected from WWTP of Frina (Table 1). 88.88% of tested raw wastewater samples (eight samples out of nine) were positive for at least one of the targeted genes (Table 1). SARS-CoV-2 RNA was also detected in three of effluent wastewater samples (n = 9) namely in Sahline and Frina WWTPs (Table 1) following a secondary treatment with activated sludge.

During the second period of our study, we have tested 31 wastewater samples which were collected from 14 different districts throughout the country. 25.80% of the analyzed samples contained SARS-CoV-2 RNA with a quantities ranging from 0.18 10^3^ copies/100 ml to 59,94 10^3^ copies/100 ml of wastewaters.

**Figure 2.**
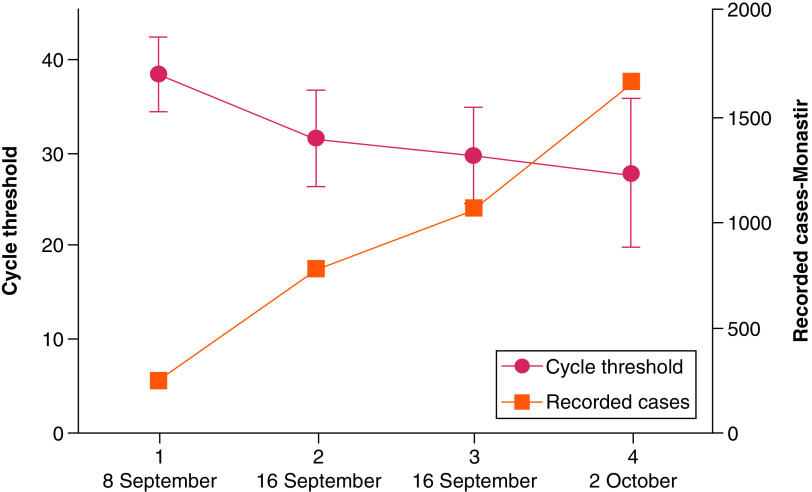
Comparison between SARS-CoV-2 RNA loads in sewage and the numbers of COVID-19 cases in the city of Monastir, 2020.

During the first period of our study, the detection of SARS-CoV-2 genetic materials in wastewaters has been combined with a rapid increase in the number of COVID-19 cases in the studied region (region of Monastir). For example, the cumulative number of reported COVID-19 cases in Monastir went from 104 cases in 8 September 2020 to 1288 cases in 2 October 2020 (data published by the Tunisian Ministry of Public Health). In parallel, the concentration of SARS-CoV-2 RNA in the sewage of each of the municipalities increased as indicated by a decrease in Ct values. The results obtained align with the pandemic surge recorded in Monastir city during the study period (Figure 2).

Concerning the other regions included in the second period of our study (February–May), clinical data, such as the number of COVID-19 cases, were not available and therefore a comparison between the number of COVID-19 cases and the concentration of SARS-CoV-2 RNA in the sewage was not possible.

## Discussion

Our results are in accordance with recent studies undertaken around the world demonstrating the presence of SARS-CoV-2 RNA in raw wastewater samples [5–20]. The number of studies that have been interested in detecting the traces (RNA) of the novel coronavirus in the wastewaters has continuously increased since the emergence of the SARS-CoV-2 pandemic. This indicates the importance of sewage surveillance as a sensitive tool for viral circulation monitoring in a population, in order to predict the appearance of epidemics and bolster the efforts deployed in the clinical epidemiology setting. Consistence between SARS-CoV-2 RNA amounts and the number of confirmed cases was observed in previous reports dealing with WBE use to monitor SARS-CoV-2 circulation in Netherlands, Australia, France, Italy, Spain, India and Japan [7–9,12,15,16,24] showing that virus monitoring in sewage is a promising tool for the surveillance of COVID-19 spread in a community. Furthermore, viral genetic materials detected are predominantly shed by asymptomatic, presymptomatic, and pauci-symptomatic carriers of COVID-19 who represent around 80% of COVID-19 infections [25]. Thus, wastewaters surveillance could be used as a tool to determine the true scale of the virus spread and thereby alert to the high presence of the virus. The number of identified cases of COVID-19 is actually the visible part of the iceberg and does not reflect the true magnitude of the virus widespread within a population (reviewed in [26]). This seems to be also valid for our study, the real number of infected people in the region of Monastir at the beginning of October 2020 (1258 cases on 2 October 2020) was actually much higher as a large portion of the population was actually asymptomatic and pauci-symptomatic that contributed to a viral transmission. This may explain the fact that few days later, the number of COVID-19 has tremendously increased and a total of 2752 cases have been recorded on 11 October 2020 (Regional Direction of Health) pushing the authorities to decree partial lockdown, ban gatherings, and strengthen health measures in Monastir region. Regarding the other regions included during the second period of our study, we were unable to obtain the number of new COVID-19 cases, hospitalizations etc.

In this context, some studies have tried to address this question by developing mathematical models based on wastewater epidemiology and that try to determine the true scale of virus circulation while taking into account asymptomatic patients [7,8]. Importantly, in our study the detection of high amounts of SARS-CoV-2 RNA in wastewaters had foreshadowed the upsurge of the epidemic which highlights the potential benefits of using wastewater surveillance as an early warning system. Our results were continuously communicated to the Ministry of Health to help in making decisions aiming to contain the COVID-19 pandemic.

Viral RNA was also present in three secondary treated wastewaters. Our findings are consistent with several previous studies reporting the presence of SARS-CoV-2 in secondary treated wastewaters [8,13,14,19,27,28]. However, other studies have reported the absence of SARS-CoV-2 RNA following a secondary treatment in WWTPs [15,18,29–31]. This discrepancy may be attributed to the difference in methods used in viral concentration and viral RNA detection among studies and/or methodologies used during the secondary treatment itself and its efficiency. Hence, a standardized method for wastewaters sampling, coronavirus concentration and detection should be used to be able to compare between studies and converge research activities in this thematic. Nevertheless, more effective methods in SARS-CoV-2 elimination from wastewaters are reported. These methods involve secondary treatments (Moving Bed Biofilm Reactor and Sequencing Batch Reactor), tertiary and advanced disinfection strategies (chlorination, ozonation, photo catalysis, advanced oxidation processes, filtration), and inactivation by heat and radiation (reviewed in [32]). Furthermore, novel, innovative, and ecological methods are also suggested to cope with the risk of SARS-CoV-2 transmission via wastewaters and sewage sludge reused in agriculture. For example, an interesting study undertaken by Ducoli *et al.* in Italy [33] recommends the incineration of sewage sludge which enables the destruction of organic micro pollutants and pathogens eventually present in the waste, but most importantly resulted ash was used as building material instead of being landfilled which brings together safety and usefulness of wastes.

## Conclusion

Our study, the first of its kind in Tunisia, is an addition to a growing body of studies undertaken around the world praising and recommending the use of wastewaters to monitor SARS-CoV-2 circulation and anticipate epidemic spread. In the current study, SARS-CoV-2 RNA was detected in raw and treated wastewaters collected from different municipalities in Tunisia. This was accompanied by an increase in the number of COVID-19 cases recorded in our country which emphasize the importance of sewage survey in SARS-CoV-2 spread tracking and anticipation.

## Future perspective

Unfortunately, we are still facing the COVID-19 pandemic. The virus is unceasingly mutating leading to the emergence of a new variants with increased transmissibility and pathogenicity. Hence, in the future we will attempt to monitor the presence of the new variants of SARS-CoV-2 in wastewaters which will provide an insight into determining high-risk zones and mitigating COVID-19 pandemic in Tunisia.

**Summary points**

- Wastewater-based epidemiology can be an efficient tool for virus circulation monitoring.
- SARS-CoV-2 RNA was detected in the wastewater treatment plants of different cities in Tunisia.
- SARS-CoV-2 RNA was detected in raw and secondary treated wastewaters.
- SARS-CoV-2 RNA amounts in wastewaters correlated with documented COVID-2019 patients.
- The research of SARS-CoV-2 genetic materials in wastewaters has contributed to virus tracking and has helped in making decisions aiming to reduce virus spread.
